# An Unusual Bloody Tumor behind the Uterus

**Published:** 1877-04

**Authors:** William H. Byford


### EXAMINER.

VOL. XXXIV.—APRIL, 1877.—No. 4.

### Original Communications.

### AN UNUSUAL BLOODY TUMOR BEHIND THE UTERUS.

BY PROF. WM. H. BYFORD.

[Clinical Lecture held at the Mercy Hospital, Oct. 23, 1876. Reported by Chas. A. Palmer, M. D.]

The patient I present to you to-day is Mrs. E. H. McC., aged 35 years, from the country. She is a small woman, of highly nervous temperament, with a history warranting the belief that she has for several years been suffering from some affection of the pelvic organs. She is the mother of several children, and her labors have not been attended with any unusual features, but from these, in consequence of a delicate constitution, she has always rallied slowly. Her menses are usually regular, but somewhat profuse, and attended with a sense of weight. She has most of the time leucorrhœa and more or less backache, but is able most of the time to attend to her household duties, which are sometimes arduous. She menstruated about the first of June last in what she considers a regular manner. She saw no bloody discharge again for five weeks, when she had a slight discharge that continued a much shorter time than usual. In about five weeks thereafter, she had a more profuse discharge, and with it pains resembling those attendant upon uterine contractions.

Since that time—which was early in September—there has been a pretty constant bloody discharge, sometimes quite free, but usually only moderate in quantity.

About the first of August her family physician examined her per vaginam, and believed she had retroversion of the uterus, and prescribed for her according to this supposition. He made another examination about the middle of September, and still considered the case one of retroversion. The first of October, after making another examination, he diagnosed a retro-uterine tumor; and about the middle of October he assured himself that it had grown to be much larger than at the last examination, and advised her to visit Chicago for further examination and counsel.

The patient informs me that, she experienced considerable nausea during the first three months, subsequent to the commencement of her menstrual irregularity. Within the last week she thinks she has felt motion similar to those experienced in her former pregnancies, and believes herself now pregnant.

Although she suffers some inconvenience from pressure upon the rectum and bladder, she is otherwise in the enjoyment of her usual health.

I examined her day before yesterday, and again yesterday, with my colleague, Dr. Roler.

The uterus is enlarged, pressed forward against the pubic bone, extends about two inches above the symphysis, and occupies the median line. The uterine sound was easily introduced into it three inches and a half. Behind the uterus and arising fully two inches above it, is a tumor with a deep and well-marked sulcus between it and the posterior wall of that organ. The tumor is tense, elastic, and as felt through the abdominal walls, regularly globular in shape.

Through the vagina the finger detects a very tense tumor strongly distending the *cul de sac* of Douglas. It is very prominent, pressing the posterior wall of the vagina forward beyond the cervix of the displaced uterus, and extending within an inch of the vaginal orifice.

Percussion of the abdominal tumor imparts a very distinct sense of fluctuation to the finger, placed upon the vaginal tumor. This effect of bimanual exploration assures us that both hands touch portions of the same tumor, and that the contents of it are at least partly fluid in their character. When the uterus is moved, the motion is imparted to the tumor and vice versa. There is no resonance upon percussion over any of the abdominal parts of the tumor. By auscultation no sound is heard except in the immediate neighborhood of the sulcus, between the tumor and the uterus, where a very fine arterial *bruit* is distinctly perceptible.

If we now sum up our observations, gentlemen, we find this much definitely made out; viz., there is a rapidly growing tumor, containing fluid occupying and strongly distending the retro-vaginal peritoneal *cul de sac*, extending into the abdomen more than half way from the pubes to the umbilicus, *moving* with the uterus, a fine arterial *bruit* between it and the uterus, and that in the person of an intelligent, observing woman who has borne children, who declares she feels fœtal movements in the tumor and believes herself pregnant.

In the absence of the true placental murmur, the sounds of the fœtal heart and the insignificant signs derived from ballottement the diagnosis is very obscure, and requires further exploration to determine.

As the tumor is growing rapidly and has begun to cause inconvenience from pressure upon the bladder and rectum, I deem it best to open it through the posterior wall of the vagina, and, if possible, remove it, or if this proves to be impracticable, to evacuate its contents and take measures to obliterate its cavity.

After being anæsthetized by ether, the patient was brought into the amphitheatre, placed upon the table on her left side, the vagina distended by Sims' speculum, and the operation was performed in the following manner; viz., commencing half an inch below the junction of the vagina and posterior surface of the cervix, an opening was made by the galvano-cautery knife in the median line of the posterior wall of the vagina, one inch and a half in length.

As soon as the sac was perforated there escaped a large quantity of maroon-colored fluid, when the tumor collapsed, so that the whole internal surface of the cyst could be easily reached by the finger introduced through the wound. Thus examined, the inner surface seemed irregular and the wall of the cyst firm and resisting.

The discharge having entirely ceased, the patient was about to be sent to her room, when Dr. Roler placed his hand above the symphysis, and manipulated with a view to ascertain the condition of the abdominal portion of the tumor.

This manœuvre, although performed with gentleness, started up an active arterial hemorrhage. An examination made it quite evident that the flow came from a source located high up in the cavity of the sac, and not from the divided edges of the vaginal wall, which was in fact firmly consolidated from the effects of heat. With a view to check it, a mixture of Monsel's solution of the persulphate of iron, one part to two of water, was thrown through a long tube into the cavity, so as to reach the more remote part of the cyst. This, however, failed to produce any effect upon the hemorrhage. The whole cavity was then filled with pellets of cotton-wool, half as large as a hen's egg, saturated with the same mixture. Each piece of cotton was secured by twine so that it might be easily removed. It was not until the sac was entirely filled and firmly packed with this prepared cotton, that the bleeding was arrested. By this time the pulse of the patient was decidedly affected by the loss of blood.

After the patient was removed from the table, the lecturer proceeded to say: The results of this operation are somewhat surprising. I had not expected to find this tumor filled with fluid blood, much less to encounter such formidable hemorrhage.

Now what is this tumor? By the light shed upon this question in the operation, notwithstanding it was filled with blood, this tumor cannot be regarded as an ordinary retro-uterine hematocele. The following, among other reasons, seem to me sufficient to justify this conclusion: 1st, It was a *growing* tumor, and I believe it had been steadily increasing in size for over four months. 2d, It moved with the uterus. 3d, It was filled with fluid blood. 4th, An arterial *bruit* could be distinctly heard. 5th, It bled actively after it was opened. 6th, The sac was firm and elastic, as proven by the resistance to the finger introduced for the purposes of exploration, after the evacuation of the fluid and the firm packing with the cotton.

By contrasting these with the conditions observed in the ordinary intra-peritoneal hematocele, we may better appreciate the significance of these propositions.

The hematocele attains its full size in a very few days. If it reaches above the uterus, it covers and surrounds that organ, is not elastic but doughy and soft; is not so well defined in its upper boundary, is not wholly movable with the uterus as this tumor was; there is no arterial *bruit*, and it is attended with much more local suffering. Again, if we puncture an hematocele several days after its formation, there issues reddish serum, and the finger introduced into its cavity meets with clots of more or less firmness, corresponding to the length of time after the effusion has taken place. Sometimes these coagula adhere so tenaciously to the walls of the adventitious cyst, that it is difficult, if not impracticable, to completely remove them.

These reasons seem to me sufficient for removing the tumor under consideration from the category of hematocele, as the term is usually considered Then the question recurs, what is the nature of this tumor and in what tissues is it located ? In answering this question, let me draw your attention to the fact pointed out by anatomists, that there is quite a considerable amount of connective tissue richly supplied with blood vessels, situated between the peritoneum and posterior part of the cervix, and extending upward somewhat on the body of the uterus, and slightly downward on the posterior wall of the vagina. I believe this is the locality of the sanguineous effusion. If we suppose that an artery passing through this mass of connective tissue, had given way and continued to inject its blood into it, we have sufficient cause for the commencement of the tumor. After this has taken place, the pressure exerted by the vis a tergo of the circulation would further distend the artificial cavity, until it might assume the formidable dimensions and present all the phenomena observed in the case before us. It might in fact become a false aneurism, and the enveloping tissues be hypertrophied to a degree corresponding to the resistance and density of this cyst. This attachment would readily account for the fact that the tumor moved with the uterus. This theory of its origin would also enable us to explain why the hemorrhage succeeded the evacuation of the contents of the tumor.

I cannot call to mind any other condition of things, by which all the circumstances attending its growth and evacuation could be intelligently accounted for.

Believing the reader would be interested in the subsequent history of the case, the reporter subjoins a brief transcript, or rather summary, of his notes after the operation. During the first twenty-four hours, two of the pieces of cotton were removed, at the end of forty-eight hours all the remaining portion of the tampon was withdrawn. This was easily accomplished by traction upon the pieces of twine attached to the several pellets of cotton. No bleeding succeeded this removal, and not the least odor could be detected, so that the iron acted both as a perfect hemostatic and disinfectant. The first and second days, the vagina was well washed out by carbolized water, and afterward the cavity of the cyst was cleansed three or four times daily by conducting the same fluid into its deepest recess by an elastic male catheter. The other treatment consisted in drawing off the urine every six hours for three or four days, giving morphia sufficient to subdue pain and administering nutritious diet. The patient recovered without any untoward symptom, and was discharged on the 15th of November, sufficiently restored to make a journey of one hundred miles to her home, five miles of which were in a carriage.

Two weeks after leaving the hospital, her husband wrote to say that Mrs. Mc. was again quite well.

